# Uneven dietary development: linking the policies and processes of globalization with the nutrition transition, obesity and diet-related chronic diseases

**DOI:** 10.1186/1744-8603-2-4

**Published:** 2006-03-28

**Authors:** Corinna Hawkes

**Affiliations:** 1 Food Consumption and Nutrition Division, International Food Policy Research Institute, Washington DC, USA

## Abstract

In a "nutrition transition", the consumption of foods high in fats and sweeteners is increasing throughout the developing world. The transition, implicated in the rapid rise of obesity and diet-related chronic diseases worldwide, is rooted in the processes of globalization. Globalization affects the nature of agri-food systems, thereby altering the quantity, type, cost and desirability of foods available for consumption. Understanding the links between globalization and the nutrition transition is therefore necessary to help policy makers develop policies, including food policies, for addressing the global burden of chronic disease. While the subject has been much discussed, tracing the specific pathways between globalization and dietary change remains a challenge.

To help address this challenge, this paper explores how one of the central mechanisms of globalization, the integration of the global marketplace, is affecting the specific diet patterns. Focusing on middle-income countries, it highlights the importance of three major processes of market integration: (I) production and trade of agricultural goods; (II) foreign direct investment in food processing and retailing; and (III) global food advertising and promotion.

The paper reveals how specific policies implemented to advance the globalization agenda account in part for some recent trends in the global diet. Agricultural production and trade policies have enabled more vegetable oil consumption; policies on foreign direct investment have facilitated higher consumption of highly-processed foods, as has global food marketing. These dietary outcomes also reflect the socioeconomic and cultural context in which these policies are operating.

An important finding is that the dynamic, competitive forces unleashed as a result of global market integration facilitates not only convergence in consumption habits (as is commonly assumed in the "Coca-Colonization" hypothesis), but adaptation to products targeted at different niche markets. This convergence-divergence duality raises the policy concern that globalization will exacerbate uneven dietary development between rich and poor. As high-income groups in developing countries accrue the benefits of a more dynamic marketplace, lower-income groups may well experience convergence towards poor quality obseogenic diets, as observed in western countries.

Global economic polices concerning agriculture, trade, investment and marketing affect what the world eats. They are therefore also global food and health policies. Health policy makers should pay greater attention to these policies in order to address some of the structural causes of obesity and diet-related chronic diseases worldwide, especially among the groups of low socioeconomic status.

## Background

In a "nutrition transition", the consumption of foods high in fats and sweeteners is increasing throughout the developing world, while the share of cereals is declining; intake of fruits and vegetables remains inadequate [1]. These poor quality diets are associated with rising rates of overweight, obesity and diet-related chronic diseases, like heart disease, diabetes and some cancers. More people now die of heart disease in developing countries than in developed, and the problem is becoming more serious among the poor [2]. Low quality diets are also associated with undernutrition in the form of micronutrient deficiency, which, in turn, lowers immunity to infectious diseases. Poor diet quality is thus associated with a dual burden of malnutrition and disease.

The dietary transitions taking place are deeply rooted in the processes of globalization. Globalization is associated with changing incomes and lifestyles. Moreover, by radically altering the nature of agri-food systems, globalization is also altering the quantity, type, cost and desirability of foods available for consumption. As Kennedy, Nantel and Shetty explain, "globalization is having a major impact on food systems around the world...[which] affect availability and access to food through changes to food production, procurement and distribution... in turn bringing about a gradual shift in food culture, with consequent changes in dietary consumption patterns and nutritional status that vary with the socio-economic strata" [3]. This latter link, between globalization, food systems, and dietary change, is the subject of this paper.

The links between globalization and diet are generally under-researched, though analysts have suggested the following mechanisms are central to the globalization/diet nexus [4-23]:

• Food trade and global sourcing

• Foreign direct investment

• Global food advertising and promotions

• Retail restructuring (notably the development of supermarkets)

• Emergence of global agribusiness and transnational food companies

• Development of global rules and institutions that govern the production, trade, distribution and marketing of food

• Urbanization

• Cultural change and influence

Yet determining the precise relationship between these mechanisms and diet quality is a challenge, as is determining their relative importance to the nutrition transition. Such challenges are a reflection of the complex and multidimensional interactions between global economics and health in general [24-30]. Different perspectives give rise to an often polarized debate about the relative merits and demerits of globalization for health [31]: some say it is mainly good for health [32,33], others that it is inherently problematic [34,35]. The reality is, as for any policy choice, that globalization is likely to bring threats and opportunities, improving health in some circumstances and damaging it in others [27,36,37].

The complexity of the interactions and the potential for gains and losses is particularly pertinent to nutrition, given nutritional problems lie along a spectrum from under- to over-nutrition. Processes of globalization operating throughout the food supply chain have different effects on different parts of the spectrum. Such processes may introduce opportunities to address undernutrition by raising incomes and cheapening food, but, in so doing, introduce risks for overnutrition. Alternatively, they may benefit under-and over-nutrition by increasing the diversity of food available for consumption. Or they may damage both by generating inequality and exclusion, making an adequate and healthy supply of food accessible only to the rich.

Comprehending these scenarios and tradeoffs is a central challenge for policy makers in a globalizing world. What will be gained and lost? And who will be the winners and losers? As Labonte [27]: points out: "Tracing the impacts of globalization on health to answer such questions can be a daunting task" (p.52). To do so, Labonte stresses the need to understand the mechanisms central to globalization. Equally, he notes it is necessary to examine the global, national, community and household contexts in which these mechanisms are operating. This is important because though the mechanisms are operating globally, their effects are context-dependent: homogenizing processes can have very heterogeneous effects. So the same globalization processes will have different outcomes for people at risk from under-nutrition relative to those at risk from over-nutrition, for urban compared with rural populations, and the poor relative to the rich.

Globalization, in other words, is a dynamic process of both mass global change and local differentiation. In dietary terms, this can be articulated as "dietary convergence" and "dietary adaptation"; each, in a seemingly contradictory unity, are part and parcel of the nutrition transition [3]. According to Kennedy, Nantel and Shetty [3], dietary convergence is "increased reliance on a narrow base of staple grains, increased consumption of meat and meat products, dairy products, edible oil, salt and sugar, and a lower intake of dietary fibre" (p.9). Indeed, analysis by the Food and Agriculture Organization suggests that diets in countries more integrated into the world economy are converging in terms of primary commodities [6]. On the other hand, dietary adaptation is "increased consumption of brand-name processed and store-bought food, an increased number of meals eaten outside the home and consumer behaviours driven by the appeal of new foods available" (p.9). Convergence, the authors argue, is driven mainly by income and price. Adaptation, in contrast, is driven by demands on time, increased exposure to advertising, availability of new foods and emergence of new food retail outlets.

This paper asks if and how the policies implemented to advance the globalization of agri-food systems are linked with the coexistence of the apparently contradictory processes of dietary convergence and adaptation in developing countries. It explores one of the central mechanisms of globalization, the integration of the global marketplace, specifically, the impacts of the three major processes of market integration on dietary patterns. The three processes are: (I) *the production and exchange of goods *in the form of agricultural production and trade; (II) *the flow of investment across borders *in the form of foreign direct investment (FDI) in food processing and retailing; and (III) *the global communication of "information" *in the form of the promotional food marketing. These processes represent important aspects of the food supply chain from production to consumption. For each of the major processes, the paper presents a case study examining the relationship between specific policy changes and dietary transitions in different contexts, focusing on the more negative aspects of dietary change and the implications for the diets of the poor.

### I. The production and exchange of goods in an integrated market place: the role of agricultural production and trade in dietary transitions

Global market integration is characterized by a combination of formerly separated markets into a single market. Agriculture is central to this aspect of globalization and the theory of comparative advantage that lies behind it: creating efficiency by locating the production of agricultural goods where there is a comparative advantage in producing them. In a globally integrated agricultural market, the idea is that nations specialize in producing food consistent with their resource endowment, and then trade those foods between themselves. The desired result is greater economic efficiency, a more consistent food supply, lower costs of production and, in theory, cheaper food.

Prior to the era of modern economic globalization, countries tended to favour the protection of domestic agricultural markets, a tendency clearly inconsistent with the economic efficiency envisioned by the theory of comparative advantage. Increasing the market-orientation (i.e. degree of liberalization) of the production and exchange of agricultural goods within and between nations has thus become a critical component of globalization. During the 1970s and '80s, many low- and middle-income countries underwent "structural adjustment," which included implementing more market-oriented agricultural policies. The pace of reform accelerated in the 1990s as many countries liberalized their agricultural markets internally and internationally. Regional trade agreements, signed at a steady but slow pace through 1970s and '80s, soared at a rate of 15 per year in the 1990s [38]. And in 1994, agriculture was included in global trade rules for the first time: the Uruguay Round of the General Agreement on Tariffs and Trade (GATT) Agreement on Agriculture pledged countries to reduce tariffs, export subsidies and domestic agricultural support. Food and agricultural trade were also affected by bilateral agreements and new rules on technical barriers to trade. This range of policy shifts over the past 20–30 years has led to a more liberal global agricultural marketplace, although it cannot yet be described as "open" since high levels of protection still exist in various forms.

This liberalizing agricultural market has enabled more and different food trade, higher foreign investment and the enlargement of transnational food companies (TFCs). In developing countries, food import bills as share of GDP more than doubled between 1974 and 2004, and the amount of trade made up of processed agricultural products rose much faster than primary agricultural products [38]. More open trade and investment have made buying companies, products and services easier across national borders, so creating incentives for TFCs to grow through global vertical integration and sourcing [39]. Global vertical integration – when a company brings together the entire process of producing, distributing and selling a particular food under its control by buying and contracting other companies and services worldwide – reduces the transaction costs associated with having different suppliers and creates economies of scale [40]. Global outsourcing – when a company searches for inputs, production sites and outputs where costs are lower and regulatory, political and social regimes favourable – enables TFCs to cut costs and helps safeguard against the uncertainty of commodity production and product sales [39].

These changes in the global agri-food system have altered the supply of foods associated with the nutrition transition. Vegetable oils are a case in point. Oil crops have been one of the most dynamic agricultural sectors in recent decades, growing at a rate of 4.1% per year between 1979 to 1999, relative to 2.1% for agriculture as a whole [6]. World oil crop production increased by over 60% between 1990 and 2003 (Table 1), with growth driven by the top three oils: soybean, palm and canola/rape. Growth has been concentrated in Asia and Latin America, not the traditional production zones of North America and western Europe. Between 1994 and 2004, edible oil production in China increased nearly two-fold, soybean oil production in Brazil by one-half and Argentina by two-fold, and palm oil production in Malaysia by two-thirds [41]. Similar trends are seen for consumption. During this time frame, vegetable oil consumption in the United States and western Europe increased by just one-quarter, whereas it doubled in China and increased by one-half in India. Overall, between 1982/84 and 2000/02, vegetable oils contributed more than any other food group to the increase of calorie availability worldwide (by 70 Kcal/capita/day) (calculated from [42]). Vegetable oils can thus clearly be implicated in rising dietary fat intakes worldwide [43]. Increased consumption can be explained in part by rising demand, but also supply side policies, as illustrated by the three largest emerging economies, Brazil, China and India, and the world's largest oil: soybean.

**Table 1:** World oilcrops primary production (Mt)

**1980**	**1990**	**1995**	**2000**	**2003**	**2004**
49,298,300	75,410,698	91,857,399	110,043,440	123,168,460	132,726,738

Source: FAOSTAT 2005 [145]

### Case Study 1: How global market integration of vegetable oil production has facilitated the globalization of consumption: the case of Brazil, China and India

Brazil is the world's second largest soybean producer and exporter (the United States is the largest producer and Argentina the largest exporter). Through the 1960s and 70s, government policies explicitly promoted production, export and domestic consumption of soybean oil [44]. In the 1990s, in line with the globalization agenda, the government opened up its soybean market and reduced government intervention. New policies reduced restrictions on foreign investment (to encourage the entry of more foreign capital into the soybean market), restructured farm income taxes (to encourage greater investment in soybean production), lowered import tariffs on fertilizers and pesticides (to facilitate higher soybean yields), and eliminated the soybean export tax (to promote greater exports) [44]. The government also implemented the "*Real *[currency] Plan" which altered the nation's economic conditions; devaluation of the *Real *later in the decade caused the cost of Brazilian beans on the world market to fall [41]. These policy changes spurred, as intended, acceleration of production and exports. Production costs fell and returns to producers rose; combined with the abundant availability of low-cost land, this encouraged farmers to bring more land into production [45]. And in light of lower production and transportation costs, vertically-integrated TFCs, such as US-based Cargill (the largest soybean exporter in Brazil) and Bunge (the largest soybean processor), increased their investments in the Brazilian crushing industry [44].

The result of these policy shifts was a 67% increase in soybean oil production between 1990 and 2001, a more than doubling of exports, and one of the lowest soybean oil prices worldwide [41] (Table 2). But somewhat ironically, the massive investment and growth in soybean oil production in the 1990s is not actually associated with increased consumption in Brazil: although the data are difficult to interpret, per capita calorie consumption (already relatively high) appeared to decline, or at least stabilize during the 1990s (Table 2). Rather, production was set for the global market, facilitating dietary changes across the globe in countries, like China and India, who were also liberalizing their markets in line with the globalization agenda.

**Table 2:** Brazil Soybean and soybean oil production, exports, and consumption.

	**1989–1991**	**2000–2002**
Soybean production (Mt)	19,629,093	37,580,396
Soybean oil production (Mt)	2,679,413	4,467,667
Soybean oil exports (Mt)	732,659	1,556,142
Calories available from soybean oil/cap/day	326	258
Calories available from all vegetable oils/cap/day	371	319
Soybean oil as percentage of calories available from all vegetable oils (%)	88	81
Urban household consumption of soybean oil (% of total daily calorie consumption)	11.4	10.1

Source: based on data from FAOSTAT 2005 [42,145,146]. The numbers represent 3 year averages around 1990 and 2001

Source of household consumption statistics: IBGE 2004 [147]. Note that FAO data suggests a clear decline in calories available for consumption of soybean oil, whereas household consumption statistics show a very slight decline in percentage consumed. The explanation of this trend is not clear. It is not clear whether the urban bias of the household consumption statistics can explain this discrepancy. Household statistics also do not account for food consumed away from home

China implemented new tax and import regulations to encourage soybean oil imports and greater domestic production in the 1990s [41]. Brazil, able to produce at low prices, became a major source in China of soybeans (for crushing) and soybean oil [46]. Between 2002–4, Brazil remained a crucial supplier of soy to China when greater trade openness led to a doubling of agricultural imports, of which soy formed a large proportion [47]. Consequently, the amount of soybean oil available for consumption in China has soared (Table 3). While this probably bought some benefits to under-consuming populations, consumption of vegetable oils in urban and some rural areas now exceeds recommended levels, a trend the Chinese government has identified as a source of concern given the rapidly rising rates of obesity and chronic diseases in the country [48,49]. Recent trade policies will likely further the ready availability of soybean oil: China's accession to the World Trade Organization (WTO) has reduced import tariffs and quantitative restrictions, which is predicted to significantly increase soybean oil imports, lower prices and increase demand [46,50,51]. Moreover, China continues to view Brazil as a good source of cheap soybeans: the Chinese government is planning to invest US$5billion in Brazilian transportation systems to help them continue to produce soybean oil at competitive prices [52].

**Table 3:** China- Soybean product imports and consumption of soybean oil.

	**1989–1991**	**2000–2002**
Imports of soybeans (Mt)	1,961,944	14,368,805
Imports of soybean oil (Mt)	435,735	736,254
Calories available from soybean oil/cap/day	27	78
Calories available from all vegetable oils/cap/day	141	213
Soybean oil as percentage of calories available from all vegetable oils (%)	19	37

Source: based on data from FAOSTAT 2005 [42,145,146]. The numbers represent 3 year averages around 1990 and 2001

India, itself the world's fifth largest producer of soybean oil, likewise imports Brazilian soybeans and oil. In the mid-1990s, India was a relatively small importer of vegetable oils; by 1998 the country had become the world's leading importer [53]. This rapid change can be directly related to market liberalization. In 1994, as part of its obligations under WTO rules, India eliminated the state monopoly on imports [53]. Facing low domestic production, imports poured in, especially of the lowest cost oils: palm and soybean oil (Table 4). Brazilian (and Argentinean) soybeans and oil were favoured owing to their lower price and transportation costs relative to the United States. Brazil also had the advantage of a high season and thus cheaper beans during the seasons of low production in India [53]. The result was lower prices for vegetable oils, increased consumption, and increased share of consumption of imported oils: by the end of the 1990s, soybean oil accounted for 21% of consumption (and palm oil at 28%) (Table 4). This stands in stark contrast to the complete dominance of consumption of peanut, rapeseed and cottonseed oil in the 1970s, a reflection of domestic production [53]. Today, prices of edible oils in India are now more affected by soybean output in Brazil, Argentina and the United States than by domestic production [54].

**Table 4:** India- Key vegetable oil statistics.

	**1989–1991**	**2000–2002**
Imports of soybeans (Mt)	102	432
Imports of soybean oil (Mt)	25,944	1,055,083
Calories available from soybean oil/cap/day	11	48
Imports of palm oil (Mt)	353,790	3,317,333
Calories available from palm oil/cap/day	7	66
Calories available from peanut, cottonseed and rapeseed oils/cap/day	107	76
Calories available from all vegetable oils/cap/day	158	231
Soybean oil as percentage of calories available from all vegetable oils (%)	7	21
Palm oil as percentage of calories available from all vegetable oils (%)	4	28

Source: based on data from FAOSTAT 2005 [42,145,146]. The numbers represent 3 year averages around 1990 and 2001

This complex web of economic globalization illustrates how a series of policy reforms in three different countries had the effect of integrating the global soybean oil market, and, in so doing, facilitated the worldwide convergence of higher soybean oil consumption worldwide. Dietary convergence has occurred not only in the use of soybean oil in cooking, but in hydrogenated form in processed foods. Hydrogenation leads to the creation of *trans *fats, which increase the risk of coronary heart disease [55]. Governments in Brazil and the other Mercosur countries, Canada and the United States have ruled accordingly that *trans *fats must be labelled on packaged foods [56]. Despite these efforts to encourage consumers to eat fewer foods containing *trans *fats and high amounts of vegetable oils, dietary convergence of soybean oil consumption is likely to continue: the WTO is expected to reach an agreement in the next few years to further liberalize the vegetable oils market [41]. Along with implications for consumption of total fat and *trans *fats, this trend introduces health concerns because it is likely to change the overall balance of fatty acids consumed in the global diet [57].

Importantly, though, the increasingly integrated nature of the soybean oil market is equally likely to facilitate dietary adaptation. The increased supply of soybean oil on the world market is leading to greater competition with alternative oils, thereby providing a bottom-line incentive for increased differentiation [41]. The process is already in evidence, with TFCs adapting soybean oil to appeal to higher-value market niches, in this case, the wealthy "health conscious consumer" wily to the detrimental health effects of *trans *fats. In September 2004, Monsanto, in partnership with Cargill, announced the development of the "Vistive™" soybean [58]. The bean will only require partial-hydrogenation during processing, thus reducing the *trans *fat content. Cargill intends to pay producers a premium for the beans, which will be past onto food processors, and eventually, as a component in processed foods, onto consumers willing to pay more for a *trans *fat-free product. In October 2004, competitor DuPont, in partnership with Bunge, also introduced a soybean with similar properties, "Nutrium™" [59]. In years to come, it is possible that leading companies will compete as much on high-priced oils for health as on low prices for the mass market; the former will encourage dietary adaptation while the latter its convergence. Thus the very same processes driving the global market integration of vegetable oils may well have very different outcomes for low- and higher-income consumers.

### Ii. The flow of investment across borders: the role of foreign direct investment in food processing and retailing in dietary transitions

Like trade, investing across borders plays a fundamental role in integrating the global marketplace. It allows companies to buy, sell and invest in other companies in other countries. One of the most important types of investment is foreign direct investment (FDI). FDI can be defined as a long-term investment by an enterprise in one country into an enterprise in another, in which the foreign enterprise becomes a foreign affiliate the parent (transnational) company. It is one of the processes through which vertical integration can take place and TFCs can grow. FDI into developing countries grew more than six-fold between 1990 and 2000, which is faster than either GDP or trade [60]. It is now the largest source of external financing for developing countries [61].

The global regulatory environment around FDI has become significantly more liberal in past decades: between 1991 and 1999, there were 1035 changes in regulations governing FDI worldwide; 94% of these changes facilitated FDI by decreasing disincentives or increasing incentives [61]. Many of the new regulations were forged in trade agreements and investment treaties: the number of bilateral investment treaties rose from 181 at the end of 1980 to 1,856 at the end of 1999 [61]. Like trade, fewer barriers and more incentives to investment enable transnational companies to cut costs, gain market power and obtain efficiencies in marketing and distribution. This has brought huge changes in the global agri-food system, as already shown by the case of vegetable oils. Back in the 1970s, the first major phase of FDI into the food supply chain focused on producing raw commodities for export, as TFCs such as Cargill and Bunge invested abroad in oilcrops and cereals for export. In the 1980s, as liberalization accelerated, FDI began to shift away from raw materials for export to processed foods for the host market, as TFCs such a PepsiCo and Nestlé invested in foreign manufacturing facilities for foods such as soft drinks, confectionary, dairy products, baked goods and snacks.

Food processing is now the most important recipient of FDI relative to other parts of the food system, and FDI is more important in the global processed foods market than trade. US FDI into foreign food processing companies grew from US$9 billion in 1980 to US$36 billion in 2000. Sales by those companies increased from US$39.2 billion in 1982 to US$150 billion in 2000 [62]. Trade, by contrast, generated a relatively small US$30 billion in processed food sales in 2000. Investments into outlets selling processed foods have also soared, especially since 1990. FDI from US-based supermarket chains grew to nearly US$13 billion in 1999, up from around US$4 billion in 1990 [62]. In 1998, US-based TFCs such as McDonald's and KFC, invested US$5.7 billion in eating and drinking places overseas [63]. While a high proportion of this FDI is still targeted at high-income countries, an increasing proportion is entering developing and transition markets, notably in Latin America, Asia and Central and Eastern Europe (see [12]).

FDI is thus playing a role in the nutrition transition by shaping the processed foods market and making more processed foods available to more people [12]. As detailed in Hawkes [12], FDI has made it possible to lower prices, open up new purchasing channels, optimize the effectiveness of marketing and advertising, and, ultimately, increase sales. The result has been a dual process of dietary convergence towards processed foods consumption (albeit not among the lowest income consumers), and dietary adaptation to a wider range of processed foods targeted at different niche markets, as illustrated well by the case of Mexico.

### Case Study 2: How foreign direct investment in the manufacture and retail of processed foods is facilitating the globalization of consumption: the case of Mexico

The globalization of the Mexican food economy is profoundly linked with its neighbour, the United States. Market integration between the two countries began in earnest in the 1980s, and was greatly accelerated by the North American Free Trade Agreement (NAFTA), signed by the United States, Mexico and Canada in 1994. NAFTA contained key agreements designed to facilitate foreign investment, including: equal treatment of domestic and foreign investors (elimination of the 49/51 rule designed to prevent foreign investors owning more than 49% of a company); prohibition of applying certain performance requirements to foreign investors (e.g. minimum amount of domestic content in production); increased rights for foreign investors to retain profits and returns from initial investments; and the prohibition of new laws that would change the status of foreign investments once established [64,65]. The provisions were applied in domestic law through the Mexican Foreign Investment Act of 1993, which repealed the previously restrictive 1973 Act to Promote Mexican Investment and Regulate Foreign Investment.

A significant (and intended) consequence of these more liberal investment rules was a rapid acceleration of FDI from the United States into Mexican food processing. The new regulations created the incentive to invest, and TFCs were attracted to the increasing purchasing power of the large, young and growing Mexican population (including a middle class), close proximity and rising urbanization. In 1999, US companies invested US$5.3 billion in Mexico's food processing industry, a 25-fold increase from US$210 million in 1987, and more than double the US$2.3 billion in the year before NAFTA [65,66]. While companies from other countries also invested, the US dominated: of the US$6.4 billion FDI in Mexican agricultural and food industries between 1999 and 2004, approximately two thirds was from the United States [64]. In 1998, sales from US food industry affiliates in Mexico exceeded US$12 billion, easily surpassing the value of US processed foods exports (US$2.8 billion) [65].

Nearly three-quarters of FDI was into the production of processed foods, and it stimulated considerable growth of the sector. Between 1995 and 2003, sales of processed foods expanded by 5–10% per year in Mexico. Recent sales growth has been particularly rapid for snacks (12% rise from January to June 2004), baked goods (55.4% rise from 2000 to 2003), and dairy products (48.1% rise from 2000 to 2003) [67,68]. Calories from carbonated soft drinks increased from 44 to 61 Kcal per capita per day between 1992 and 2000 [69]. Consumption of Coca-Cola drinks (mainly Coke) rose from 275 8oz servings per person per year in 1992 to 487 servings in 2002 (greater than the 436 servings per person in the United States) [70,71]. Eating "comidas chatarras" ("junk food") in general is very common among children in parts of the country [72]. Even in rural areas, it is typical for children to buy soft drinks and snacks everyday in school breaks [73]. Higher consumption of these energy-dense foods is thought to be associated with increased consumption of dietary fats and sugars in Mexico, which has in turn been linked with obesity and diet-related chronic diseases [74-77] (see Table 6). Concern about obesity and diabetes is in fact leading to a counter-trend in the processed foods market: increased sales of "diet" foods. [78,79]. Coca-Cola, for example, introduced 20 new "health" drinks in Mexico in 2005, which market analysts say reflects the company's fear that concern about diabetes could reduce soft drink consumption [80].

A second affect of NAFTA on the processed foods market was to stimulate the growth of multi-national retailers [81]. The elimination of the 49/51 formula was a particularly powerful incentive, encouraging multi-nationals to create alliances with existing domestic companies, and then buy them out [82]. The result was an "explosive" growth of chain supermarkets, discounters, and convenience stores, from less than 700 to 3,850 in 1997, and 5729 in 2004 [67,83]. US-based Wal-Mart de Mexico (known as Walmex) was particularly successful, and is now the nation's leading retailer (Table 5). In the 2000s, growth of convenience store chains (stores selling a limited number of convenience items 24 hours a day) surpassed the supermarkets. Market leader, OXXO (owned by Coca-Cola subsidiary, Femsa), tripled its number of stores to 3,500 between 1999 and 2004, while 7-Eleven doubled its number of stores between 1999 and 2004 to 500, and plans to double in size again in the next few years [84,85]. Overall, sales from convenience stores increased by 17% in 2004 [86].

**Table 5:** The success of Wal-Mart de Mexico (Walmex): key facts.

• Walmex is Mexico's leading retailer, with 420 supermarkets and discount stores, and 290 restaurants, in 79 cities
• Walmex stores sell food, general merchandise and food; as a percentage of total sales, general merchandise and clothes actually make up more than food
• There were 663 million customer transactions at Walmex in 2004
• Sales from Walmex have grown rapidly over the past decade; in 2004, sales increased by 11% to reach a record high of US$12.4 billion
• Walmex continues to grow: In 2005, it invested US$625 million to open a further 77 outlets
• Reflecting their aggressive stance on low prices, Walmex's tagline is "low prices everyday"
• The company employs more people (109,075) than any other company in Mexico
• Walmex's success has left its three main Mexican supermarket rivals struggling to compete, and is attributed with causing the French supermarket giant, Carrefour, to withdraw from the Mexican market in 2005

Sources: [67,89,148,149].

**Table 6:** Food consumption, obesity and diet-related chronic diseases in Mexico.

Between 1988 and 1999, percentage of total energy intake from fat increased from 23.5% to 30.3% and between 1984 and 1998, purchases of refined carbohydrates increased by 37.2% [77,150]. Although the absolute increases of fat were higher in the wealthier north and Mexico City (30–32%), the poorer southern region also experienced a significant increase (22%). At the same time, trends in obesity and diabetes are reaching "epidemic" proportions. Overweight/obesity increased 78% between 1988 and 1998, from 33% to 59% [150]. Obesity is now quite high in some poor rural communities [151]: the greatest relative changes occurred in the poorer southern region (81%) compared to the wealthier north (46%). More recent figures estimated overweight/obesity at 62.5% in 2004. While the obese clearly consume sufficient energy, the same cannot be said of micronutrients: women who are underweight, normal weight or overweight/obese are equally likely to suffer from anaemia [152]. Obesity is also giving rise to an epidemic of diabetes which is rising fastest in the poor regions [153]. Over 8% of Mexicans now have diabetes, which the WHO estimates costs the country US$15 billion a year [154,155].

By 2004, in just a decade since NAFTA, supermarkets, discounters and convenience stores, accounted for 55% of all food retail in Mexico, and now dominate the sector in large and medium-sized cities [84]. The remaining 45% of food retail comprises thousands of traditional "tiendas" (small, family-owned, general merchandise stores or street vendors) and open markets. Tiendas are still the most important form of food retailing in small towns and rural areas, and cater in general to low-income populations. In 2003, tiendas accounted for over 90% of food purchases in small towns, compared with less than 30% in towns with populations over 250,000 [85].

Food retailing has played an important role in the expansion of the processed foods market in Mexico. Tiendas have proved critical to the spread of "comidas chatarras"; they are the means by which transnational and domestic food companies sell and promote their foods to poorer populations in small towns and rural communities. Over 90% of all Coca-Cola and PepsiCo sales are, for example, from tiendas. Coca-Cola has a formidable system of distribution to thousands of tiendas all over the country, actively encouraging owners to stock their drinks by providing free incentives, such as point-of-sale materials and refrigerators, in return for an exclusivity agreement. (In November 2005, Mexico's Federal Competition Commission upheld a fine levied against Coca-Cola Femsa for allegedly pressuring shopkeepers in tiendas not to offer another cola brand at their stores. The ruling will have the affect of making it easier for tiendas to sell cheaper, B-brand colas, such as "Big Cola", owned by a Peruvian company.) PepsiCo also distributes through a tienda network, using the stores for sales promotions linking their soft drink and snack food brands [73].

The growth of convenience store chains has had the affect of injecting further dynamism into the market for "comidas chatarras". Convenience stores typically sell hot and cold snacks, such as microwaveable products, donuts, ice cream and soft drinks. Usually located in more affluent neighbourhoods, they mainly target urbanites with limited time wanting a quick snack. The stores have helped grow the processed food market by being easily accessible (open 24 hours) and, despite limited stock, being well-positioned to sell higher-priced, more novel processed foods (with higher profit margins) [85]. As the number of convenience stores increases, it is likely that they will compete more aggressively with tiendas to broaden their customer base. The amount of food purchased from tiendas is declining year-by-year as they close in the face of competition from other retailers, particularly convenience stores: according to the Mexican Chamber of Commerce, five tiendas close for every convenience store that opens [85].

The growth of supermarkets and large discount stores has even more important implications for the long-term expansion of the processed foods sector. Over the long-term, the market for processed foods grows through segmentation, which involves the development of new products targeting different market niches to activate and reactivate demand in a changing consumption environment [87]. Supermarkets are ideally placed to deliver the adaptive tendencies of this market dynamic. Through their size and capital base, supermarkets are able to make available a far wider range of processed foods than tiendas and convenience stores, and to take the risks inherent in introducing new foods. Due to economies of scale in storage and distribution and technological advancements in supply logistics, supermarkets are able to sell processed foods at lower prices, while still maintaining profits [88]. Consequently, supermarkets are able to frequently update their stock to create and adapt to demand, thereby delivering (and encouraging) the market segmentation strategy of the processed foods industry. Delivering recent innovations in the "diet" foods market is a case in point: to target the more affluent, health-conscious niche, Walmex now stocks over 250 diet products, including low-carb chocolate and sugar-free candy, and reports that consumer spending on such products is increasing [78]. Sales of these relatively high-priced diet foods rose in Mexico by 20% in 2003, a rate that is expected to continue [79]. In contrast, the very same supermarkets also manage their stock to attract the lower-income, budget conscious niche: increasing shelf space for cheaper, private label goods and "B-brands"; introducing smaller pack sizes, which although more expensive per unit, are more affordable because of their lower price [84]. These dynamics have interesting dietary implications. Increased variety and segmentation could have the positive impact of improving the availability of "healthy choices" (a dynamic which would also affect fresh foods such as fruits and vegetables): Wal-Mart de Mexico actually assert that the variety of food they offer at low prices increases the possibility of "improving the population's diet" [89]. Or the strategy could simply expand the market for energy-dense, nutrient-poor foods, or, perhaps most likely, the development of different dietary habits between different groups.

FDI has fostered much of the growth of processed foods and modern retailing in Mexico, either directly by increasing the size of the market, or indirectly by stimulating competition with domestic firms. The profit-seeking nature of the more open processed foods market has encouraged growth and segmentation. FDI in manufacture and retail has been complementary: the success of the growth and segmentation strategy of the processed foods industry has required the presence of different types of retailers, and is now being delivered over the long-term by the supermarkets. One of the central processes of global market integration, FDI and the policies that encourage it, is thus facilitating dietary convergence towards consumption but also adaptation to dietary niches. If this dynamic continues, the process of convergence could well lead to very divergent dietary outcomes between rich and poor.

### Iii. The global communication of "information": the role of food advertising and promotion in dietary transitions

Owing to its visibility, promotional food marketing ("marketing") has become one of the hallmarks of globalization. Coca-Cola signs, ubiquitous in countries around the world, are a classic symbol of what is often assumed to be the homogenous nature of globalization. The intended impact of marketing on food consumption is also quite apparent (a great deal more so than trade or FDI). Marketing explicitly involves designing strategies and implementing activities to influence consumption habits and create demand. It involves not just advertising, but a whole array of methods including sales promotions, websites, viral marketing, music and sports sponsorship, product placement in films and television, and in-school marketing. TFCs, and the advertising and marketing agencies that serve them, use these techniques to encourage more people to consume the product, more frequent consumption among people already familiar with the product, and consumption of more of the product at one time [90]. Food advertising and promotion is now a global phenomenon, occurring in even remote parts of the world [90]. During the period 1980 to 2004, global advertising expenditure rose from US$216 billion to US$512 billion [91]. Promotions for energy-dense, highly-processed foods aggressively target young people, aiming to influence food consumption patterns that will carry into adulthood. In Western countries at least, such advertising has been shown to influence dietary habits among children [92,93].

Marketing is more than just a visible and tangible form of globalization. It is also, like trade and FDI, a *process of *globalization. Marketing speeds the flow of food products spread by trade and FDI into the global marketplace: In a larger, more dynamic marketplace, companies benefit from rapid product turnover, and marketing speeds up this process. It does this by attracting attention to new products, creating perceived differences between similar products, and improving the apparent value and desirability of products. In so doing, marketing encourages more consumers to consume the products, and more producers to produce them, thus advancing the cycle of global market exchange and integration.

It is a self-reinforcing process: just as marketing facilitates globalization, globalization facilitates marketing. Globalization brought to the developing world the advertising/marketing agencies with the most expertise in designing marketing campaigns. From the 1980s onwards, advertising agencies transnationalized and consolidated through FDI, mergers and acquisitions, growing into huge, vertically integrated global corporations [94,95]. The process was driven by a range of incentives: the companies which commission the services of marketing agencies were transnationalizing, as were the media networks they utilize; communications technologies were improving; the market for communications services was becoming more open due to some domestic deregulation and trade agreements; and prospects of higher profits and revenue growth were greater overseas [95-97]. Today, just a handful of communications networks control most of the global market. Though, mainly headquartered in the United States, Europe or Japan, networks and agencies have hundreds of local offices worldwide. An important outcome of this global consolidation was that agencies previously concerned solely with advertising bought in additional expertise in non-media advertising, market research, and communications services. This allowed them to supply to their clients co-ordinated and comprehensive campaigns encompassing a wide range of promotional techniques, from advertising to direct mail, from school-based marketing to sports sponsorship deals [94]. Globalization also enabled the spread of technologies that introduce more places to advertise. Television ownership spread rapidly through the developing world during the last decades of the 20^th ^century, accompanied in the 1990s by the market liberalization of public television and subsequent increase in commercial programming [98]. More recently, technological development further broadened global communication networks, notably through the Internet and phone networks [97].

The globalization of food marketing thus comprises three core components: the globalization of TFCs and the foods they promote; the globalization of advertising/marketing agencies; and the globalization of communication technologies. Together, they have increased the power of marketing as an agent of dietary change, as well-illustrated by food marketing in Thailand.

### Case Study 3: How global market integration of food marketing has facilitated the globalization of snack consumption: the case of Thailand

The advertising and promotions industry in Thailand is among the most developed, dynamic and "creative" in the region [99-102]. From 1987 to 1996, advertising expenditures grew nearly 800%, and advertising revenues have grown at double digit figures in recent years, standing at around Bt85 billion (US$2.0 billion) in 2004 (note all currency equivalents use December 2005 exchange rates and are therefore not a precise representation of changes over time) [102,103]. Promotional activities are known for their zany and humorous style. Two sets of policies have contributed to this dynamism, both related to the country's tradition of openness to trade and investment. First, foreign ownership of advertising/marketing agencies is not restricted (as it is, say, in Vietnam), and while advertising is regulated to some degree, campaigns are not subject to restrictions like maximum foreign content requirements (as they are, for example, in Malaysia) [104]. Second, free trade agreements (such as the GATT/WTO framework and the ASEAN Free Trade Area) have encouraged the influx of foreign brands (including many food brands), creating the incentive to promote differentiation between brands and products within and between domestic and multinational companies.

This relatively open market has enabled the convergence of the three core components of global food marketing. First, TFCs have entered Thailand and used a wide variety of promotional techniques to increase sales. The role of marketing in snack consumption has been particularly important (see Table 7). Unlike non-traditional processed foods like ice cream and burgers, sold mainly in wealthier urban areas, eating processed savoury and sweet snacks is common throughout Thailand, particularly among young people [105,106]. It has been reported that Thai children obtain 23% of their calories (almost one quarter) from snacks [107]. According to the market research organization, Euromonitor, snack sales "are being driven by targeting youngsters between 5 and 24 years old. Competition is becoming more and more aggressive between the key players, with advertising and promotional campaigns trying to attract consumers" [105]. A survey conducted in 2004 concluded that the major contributing factor to high-snack consumption among children was the influence of television commercials. Television is a major channel for advertising: The same survey counted an average of 67 different snack products advertised to children during weekend morning television (7 am-10.30 am) [108].

**Table 7:** The processed snack market in Thailand.

**Figure 1:**
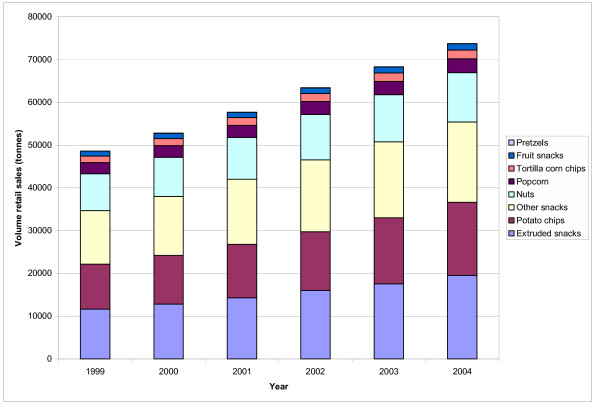
Retail sales of sweet and savoury snacks in Thailand. Source: Data from [105]

• Between 1999 and 2004, sales of sweet and savoury snacks grew by 35.2% in volume from 48,516 tonnes to 73,740 tonnes (see figure 1), and in value from Bt9 billion (US$220 million) to Bt16 billion (US$391 million) [105].
• The largest snack category is "extruded snacks" (shapes formed from potato granules, wheat flour, tapioca starch and corn starch); estimated sales were Bt5.1 billion (US$125 million) in 2004. The second largest category is potato chips, with Bt4.3 billion (US$105 million) in sales in 2004.
• The largest snack category is "extruded snacks" (shapes formed from potato granules, wheat flour, tapioca starch and corn starch); estimated sales were Bt5.1 billion (US$125 million) in 2004. The second largest category is potato chips, with Bt4.3 billion (US$105 million) in sales in 2004.
• Market leaders are both US-based TFCs: Frito-Lay Thailand Co Ltd (a division of PepsiCo) (30% share, as of 2003) and Proctor and Gamble Manufacturing Thailand Ltd (13% share as of 2003). "Lay's" is the number one brand, with a value share of 21% in 2003 [105].
• Despite the presence of major market leaders, there are in fact thousands of processed foods manufacturers: in 2003 there were around 2000. There are also hundreds of brands (for example, an estimated 360 brands of salty snacks in 2002).
• According to a survey conducted in 2004, young people aged between 5–24 years spend Bt161 billion (US$3.9 billion) annually on snacks [108]. Market research organization, Euromonitor, note that this is ten times more than their estimation, but attribute this to the high sales of unpackaged and unbranded sweet and savoury snacks [105]

Through their company Frito-Lay, PepsiCo is the single largest player in the Thai snacks market (Table 7). PepsiCo first entered the market through a joint venture in 1985, established Pepsi Foods Thailand in 1995 (renamed Frito-Lay Thailand in 1996), purchased a major rival in 1999, and, given the fast-paced nature of the market, moved their Asia-Pacific headquarters to the country in 2000 [109,110]. When Frito-Lay first entered the country, there was already a large number of domestic manufacturers of the popular "extruded snacks" which had sprung up in the 1970s and 80s (see Table 7) [111]. But keenly aware that per capita snack consumption was still relatively low (1 kg per person per year in 1999 compared with 3 kg in Mexico and 10 kg in the United States), the company developed an aggressive strategy to increase consumption [112]. Core components included introducing potato chips (previously not a major snack), creating innovation in the extruded snacks market (in part by creating alliances with existing local manufacturers), and developing new "Thai-oriented" flavours to appeal to local tastes [113-116]. Marketing was central to this strategy. In order to generate and maintain interest in their new products, Frito-Lay more than doubled their promotional spending between 1999 and 2003 (see Table 7), redesigned packaging, and developed campaigns involving a wide array of promotional techniques (described in Table 8). Campaigns were carefully tailored to target different groups in order, according to the managing director of Frito-Lay Thailand, to "keep our customers in the long-term" [117]. Advertising for Cheeto's prawn crackers focused on 6–14 year olds; Lay's promotions targeted older, wealthier people in urban areas; the Mun Mun brand (originally a Thai company) was targeted at more price conscious consumers in the more rural north [117,118].

**Table 8:** Examples of Frito-Lay marketing strategies in Thailand, 1999–2003.

**1999 **- Total annual marketing budget estimated at Bt170–180 million (USD4.2–4.4 million) [112] - Budgeted Bt45 million to promote new Doritos, targeting 15–24 year olds with adverts featuring model and MTV VJ Sonia Cooling and distributing two million free samples. The promotions aim to find "mostly new customers" for Doritos rather than just switching from other brands [156]. - Formed marketing alliance with Major Cineplex to promote Frito-Lay products in conjunction with Start Wars I [157]
**2000 **- Marketing budget "at least" Bt200 million (USD4.8 million) [118] - Launched extruded snack brand "Tawan" in alliance for local manufacturers to compete in provincial Thailand [116] - Formed a strategic alliance with Nokia (Thailand) to target Doritos at new customers. Consumers who collect four jigsaw pieces to make up an image of a cell phone received a Nokia phone. The promotion cost Bt 40 million [122]
**2002 **- Frito-Lay announced they would double their spending on promotional marketing to Bt 400 million (USD10 million) [158] - Introduction of larger snack packets offering 20% more content but with no increase in price, and offering three packets for the price of two [115] - Package redesign for Doritos
**2003 **- Launch a new flavour "Tawan larb" to appeal to "provincial consumers" promoted with a Bt50 million advertising campaign featuring Thai actress singer Pornchita "Benz" Na Songkhla [114]. - Launched Lay's Nori seaweed spending Bt200 million on promotion using British-Thai actress Kathaleeya McIntosh, chosen because of her "look-good" image [113,159,160]. - Launched new Lays potato chips: Lay's Siam Classic spending Bt50 million to promote the product including TV commercials, radio spots, magazine ads, cinema ads, sales promotional materials such as posters, and free samples [161] Aim was to "widen its customer base from teenagers to consumers aged 18–45 years [159].

Frito-Lay's strategy proved successful. Their share of the total snack market grew from the low single digits in the mid 1990s to 30% by 2003 [105], and sales increased from Bt885 million (US$21.6 million) in 1997 to Bt2865 million (US$70.0 million) in 2002 [119]. The entry of Frito-Lay into the market also had the affect of stimulating competition, thereby growing total snack sales. After Frito-Lay introduced their prawn cracker brand in 1997, promotions among domestic manufacturers intensified [120]. After Frito-Lay's success in potato chips, domestic snack maker Berli Jucker adopted deliberately similar marketing tactics, spending Bt100 million (US$2.4 million) on TV commercials and sport sponsorship in 2003, a move credited with boosting sales by 57% [121-123]. Snacks sales grew particularly rapidly during 1999–2004, the period of most intensive marketing, and sales volumes of most heavily promoted products (chips and extruded snacks) increased by the largest amount (63% and 69% respectively) [105] (Table 8, Figure 1).

Frito-Lay and their rivals were aided in their efforts by the second core component of globalized food marketing: multinational marketing agencies. The Thai advertising market has in fact been dominated by US-based agencies since the 1940s, but the rapid increase in the entry of transnational agencies marked out the 1980s onwards [102]. The major marketing agency for Frito-Lay major is BBDO Bangkok, part of the US-based BBDO Network, the third largest agency network worldwide by revenues (US$1.3 billion in 2004), with 345 offices in 76 countries [124,125]. BBDO is itself part of Omnicom media, the world's number one "corporate media services conglomerate" which came together in a mega-merger in the 1980s. Frito Lay is one of BBDO's key clients in Thailand; they are also a key client worldwide. Through this globalized alliance, BBDO have been able to bring their international experience of successful snack marketing to Thailand, and then blend it with local knowledge to create successful promotional campaigns [126]. According to media analysts, "BBDO Worldwide's international network offers Pepsi-Cola International organisational capabilities and media buying efficiencies, while supporting continued international growth across the entire Pepsi brand portfolio" [127].

The third prong in the globalization of food marketing is the spread of more places to promote products. As already described, television is a major means of marketing in Thailand. Unlike many middle income countries, television ownership is widespread: a cross-sectional national household survey conducted in the late 1990s found that 94.5% of Thai children surveyed watched television [128,129]. Even very low-income families who cannot afford to buy televisions watch it as a communal activity in cafes and food stalls. High television ownership has not, however, been driven primarily by the economic globalization of the 1980s and onwards. Television was actually introduced with support from the Thai Royal Family and Bangkok elites in 1955 – decades before other developing countries and at a similar time, or even earlier, than some high-income countries [130]. Still now, all channels are controlled by the government, and Thai broadcasting laws prevent foreign ownership of terrestrial stations. Television networks are operated by commercial franchisees on behalf of government agencies like the Royal Thai Army. The Thai government has specifically encouraged the spread of these stations throughout the country as a form of nation building and has always permitted advertising in order to finance the TV stations (unlike state-owned channels in many other countries) [130]. The ubiquity of TVs in Thailand has thus been less a process of globalization, than a context in which the globalization of TFCs and marketing agencies has flourished. Still, more recent technological developments, such as mobile phones, have arisen from the global integration of technology markets. Thai mobile phone companies have rolled out networks throughout the country, and while concentrated in Bangkok, owning a mobile phone is now common throughout the country for middle class families: it is therefore not surprising that they are now used for marketing activities (see Table 8).

The snack market in Thailand, comprising TFCs, domestic firms, and tiny family businesses, is highly competitive. Advertising and other promotional activities have helped boost the desirability and sales of particular *brands *within this market, and of the entire *category *(i.e. snacks). In what is termed "glocal" marketing, campaigns have been developed according to global objectives – increased sales and profits – but implemented locally, their medium and message adapted to multiple and diverse audiences, bearing in mind local conditions and cultures [90,102]. The same applies to the snacks themselves. By targeting different markets, TFCs move towards their global goal of increasing convergence of snack consumption towards developed country levels, albeit a goal still far from reached in much of the world [131]. As elsewhere, the dynamism and effectiveness of promotional marketing in Thailand has led to concerns that it is encouraging poor quality diets in young people and contributing to obesity, which is rising fast, even in the more rural northern part of the country [132,133]. The government has already restricted advertising of alcohol and energy drinks [134], and in 2004 met with advertising representatives and NGOs to discuss the banning of food advertisements on TV that target children aged 5–16 through "prize draws, freebies and discounts" [108,135]. Yet as long as the fundamental and structural forces driving global market integration needs marketing, marketing will continue to seek new targets, probably by increasing the amount of the less tangible "below-the-line" techniques [134]. A complement to regulation is for TFCs to actively promote healthier products and cease using marketing techniques which promote unhealthy eating habits. Frito Lay has in fact pledged a "commitment to health and wellness", but it remains to be seen if and how this commitment will be realized in Thailand [136].

## Conclusion and policy implications

### Policies and institutions in global dietary change

#### Policies designed to integrate the global food market matter for what people eat

This paper has traced the links from specific policies (or, rather, combinations of policies) in specific countries to specific changes in dietary habits. It has shown how important the policies and processes designed to advance the globalization of the world economy can be in shaping the nature of dietary change. Changing consumer incomes, behaviours and desires are clearly also important; it is when these changes converge with the macro structural forces that dietary shifts take place [137]. Policies designed to integrate the global food market – on agriculture, trade, FDI and promotional marketing – have been developed in the economic sphere, yet influence food consumption patterns. They are therefore not just global economic policies, but global food and global health policies.

#### Transnational food companies affect dietary change directly and indirectly

TFCs are key institutions driving the integration of world food markets. They produce, sell and promote products according to incentives created by policies and economics as well as consumer behaviour. TFCs affect dietary habits *directly *through producing, manufacturing, retailing and promoting different foods eaten in different countries. Public attention has tended to focus on the highly-processed foods manufactured by TFCs – and the example of Mexico shows that these products can be widely consumed. Yet in most countries, many highly-processed foods are still largely consumed by more affluent groups in urban areas [131]. Thus at the moment, TFCs are probably playing a more important role in dietary change *indirectly*, by altering the parameters of the domestic food markets. Importantly, they stimulate competition while simultaneously dominating product sectors, which alters the food market as a whole. They also create a cultural identity for different foods and introduce new ways to sell and promote them.

#### The effect of policies and institutions is mediated by the existing resources, services and technologies available

Existing resources, services and technologies and services have a major influence on the outcomes of global and national economic policies (and, indeed, influence their design). As shown here, policies designed to promote domestic production and global consumption of Brazilian soybean oil were only possible in the context of an abundant and cheap supply of land and reduced transportation costs; policies on FDI into processed foods manufacturing in Mexico paid off well in part because of the existence of traditional forms of retailing; in Thailand, globalized marketing strategies were nationally effective, in large part because of historical patterns of TV ownership.

### The convergence-divergence model of dietary change

#### Globalization influences dietary differentiation as well as convergence

Globalization is often viewed as "coca-colonization" or "McDonaldization" – a homogeneous process with homogeneous outcomes. But this paper has shown that the dynamic, competitive forces unleashed as a result of global market integration produce both convergent and divergent dietary outcomes. All three case studies show how market integration increases the incentive for TFCs to sell cheap and/or standardized food around the world, while *simultaneously *increasing the incentive to create market niches. The creation of similarity and difference is thus part and parcel of the same process – the logical functioning of the global marketplace. In dietary terms, to follow the examples given here, this means that more people eat more soybean oil and processed foods, but *different types *of people eat *different types *of these foods bought from *different types *of stores and (possibly) influenced by *different types *of marketing techniques. This convergence-divergence model unites the apparently contradictory observations that, on the one hand, global market integration homogenises diets and, on the other, brings greater food variety (since it does both). It indicates, too, that the "nutrition transition" model of dietary change – while apt and appropriate in nutritional terms – fails to capture some of the more complex aspects of global dietary change (see also discussion by Lang and Rayner [138].

#### Globalization could be encouraging the uneven development of new dietary habits between rich and poor

It has been argued elsewhere that the increased differentiation brought by globalization promotes better diet quality through increasing access to dietary diversity [20]. The same could be said of urbanization. Following this argument, the problem of obesity becomes one of diet quantity (people eating too much of a wide variety of nutritious foods) not quality (people eating a diet dominated by nutrient poor, energy-dense foods).

Yet the convergence-divergence duality raises the policy concern that globalization could be encouraging the uneven development of new dietary habits between rich and poor. As high-income groups in developing countries accrue the benefits of a more dynamic marketplace, lower-income groups may experience convergence towards poor quality obseogenic diets, as has been observed in western countries. People of low socioeconomic status (SES) (albeit not the poorest of the poor) are more likely to be influenced – over the long-term – by the converging trends of the global marketplace: the economic and cultural convergence towards cheap vegetable oils, *trans*-fats, and imitations of heavily promoted products whose desirability has been stimulated by their earlier popularity among wealthier groups. Meanwhile, the more affluent and educated move onto the more expensive, "healthy market" niches such as the *trans *fat free vegetable oils and "diet" foods.

#### The influence of globalization policies on dietary patterns is context specific

The divergent nature of the dietary outcomes of globalization is also a result of regional, national and local contexts. National socioeconomic bifurcation and the cultural context are particularly important [27]. In high-income countries, the prevalence of poor quality diets, obesity and diet-related chronic diseases tends to be higher among groups of lower SES. Unfortunately, this trend is now also beginning to emerge in middle-income countries: a recent review of the evidence concluded that as gross national product (GNP) increases in developing countries, the burden of obesity tends to shift towards groups of lower SES; after countries have crossed over a GNP threshold of about US$2500 per capita, women of low SES tend to have proportionally higher rates of obesity [139]. In other words, obesity starts out as a problem among groups of higher SES, but as national economies grow, the risk moves towards groups of lower SES. The explanation for this long-term uneven development of obesity has already been placed in the context of the convergence-divergence duality. But this duality is, of course, feeding off existing socioeconomic inequalities. In his pioneering work in Brazil, Carlos Monteiro shows a strong inverse relationship between obesity and education (not income) in women indicating an important association between education and nutritional knowledge [140,141]. Poor diets and obesity are emerging in this socio-economic context.

Culture is another important context. In the Brazilian case, a culture of "thinness" exists in more highly-educated groups, no doubt reinforcing the role of education in this particular country context; the opposite is true in other cultures. The cultural context is also important because it affects the degree of acceptability of new products and services introduced through the globalization process. This is particularly relevant for promotional activities. In an apparently contradictory process, the "glocal" marketing strategies adopted by TFCs and domestic firms often deliberately appeal to *existing *cultural viewpoints or traditions in order to *change *cultural norms and rules about what to eat, how, where and how much [90,138]. This is the true power of marketing and indicates the importance of "cultural transition" in dietary change [138].

Between them, the processes of differentiation combined with convergence, the differences between rich and poor, and the role of the socioeconomic and cultural contexts, comprise a "convergence-divergence model" of dietary change, rather than a simple transition.

### Policy implications

These conclusions present some clear implications for policies needed to address poor diets, obesity and diet-related chronic diseases. First, policies must take into account the influence of the policies and processes of global market integration on long-term dietary change, and the context in which they operate. Such a process requires looking beyond the health sector as narrowly defined, and entering into debates and policy arenas dealt with by other sectors and disciplines. Second, policies must address, in some way, the behaviour of TFCs, preferably by creating incentives to improve "healthy" market functioning. Third, policies need to focus on the promotion of healthy diets over the long-term among groups of low SES. The concern of this paper has been groups with access to diets sufficient in energy. But diet quality is important for those at risk from undernutrition; policies that focus on diet quality are therefore important for addressing problems across the whole nutritional spectrum.

Thus far, there are few comprehensive sets of policies addressing obesity and diet-related chronic diseases in the developing world. This may begin to change following the passage of the World Health Organization's Global Strategy on Diet, Physical Activity and Health in 2004 [142]. But even in high-income countries, policies still tend to focus on consumer behaviour – there is reluctance to tackle the more structural drivers of change. This partly reflects the misunderstandings around chronic diseases (especially that they are completely freely acquired), the lack of evidence in the hands of policy makers, and the low capacity for policy development [143]. But it also reflects the fact that implementing such policies necessitates confronting the powerful forces and institutions of the global marketplace – which governments actually often want to strengthen as a means of creating wealth. This is doubly a challenge because health can benefit from wealth: higher GNPs are associated with higher life expectancies. Policies are thus needed to promote *healthier *economic development.

Two commonly proposed strategies are nutrition labelling and regulating food marketing practices [56,134]. Labelling is probably the most widely utilized population-level policy, and has potential: dietary adaptation shows that consumers do have real power in the modern food system, and can be responsive to information. In turn, this can be a powerful incentive for TFCs to change their products. A real concern here, though, is that the benefits of policies based on the provision of information may accrue mainly to groups already more educated about nutrition, with all the implications for unequal dietary development.

Restricting food advertising and promotion could also alter signals to consumers and encourage product changes. Importantly, it would have the affect of creating a more supportive environment for health promotion efforts. Equally, marketing could be used more effectively to promote healthier foods, a strategy that has delivered some success through supermarkets and other points-of-purchase [144]. The concern here is that marketing regulations not only have to confront TFCs, the advertising agencies, and new technologies, but the long line of incentives in agriculture, trade and investment behind the food entering the market in the first place. Thus policies which are relatively close to the consumer, such as labelling and marketing, are worthwhile, but prone to being undermined.

To alter the series of incentives in the global marketplace from farm to fork, there is needed for policy to effect change closer to the point of production are needed. FDI is a case in point. FDI represents a single, upstream, entry point to many of the dynamics influencing the production, sale and promotion of foods in the global market place, and thus could be an effective lever for change [12]. The benefits of such approaches are that they influence markets, not just the products sold in markets. Relatively small changes at a macro-scale can also have relatively large population-wide impacts. Perhaps most importantly, they are the approaches that are most likely to benefit groups of low socioeconomic status over the long-term.
